# Extraordinary Madness

**Published:** 1849-11

**Authors:** 


*Extraordinary Madness.*—Physiological pathologists have of late been as much on the alert, in France, concerning the case of a sergeant of the line, as they have been, in this country, concerning Miss Nottidge. The two cases bear, however, no analogy to each other. Religious monomania is not rare; but the derangement of mind, leading to the frightful and disgusting acts of Sergeant Bertrand, is, as far as we can remember, perfectly unique in the annals of mental alienation. His mania consisted in exhuming the dead, and taking pleasure in mutilating the corpses; but, shocking to relate, there was an erotic tendency mixed up with these horrible deeds, and he took especial delight in raising the corpses of females, and satisfying his unnatural appetites upon their putrefying remains.

From the trial which lately took place in Paris, before a court martial, and from the confession written by himself, we learn that this unfortunate individual is twenty-five years of age. He first studied for the church, but suddenly enlisted, and, by his good conduct, obtained the rank of sergeant. When young, he was rather of a sullen and melancholy disposition, but nothing positively pointing to derangement was then observed. His hideous propensities appeared only in February, 1847, when they were excited by the sight of a grave left unfilled after interment, the diggers having been compelled to desist by a heavy shower of rain. He then struck the corpse, which he had exhumed with the tools left by the grave, with the utmost fury; and being interrupted, fled to a neighboring wood, where, according to him, he remained for three hours in a state of perfect insensibility, after having been most violently excited.

From this time to the 15th of March, 1849, this wretched man desecrated burying-places eight or ten times, both by day and night, regardless of the severity of the weather, the dangers he was encountering on the part of the keepers, and the difficulties he had to surmount. By the aid of his small sword he used to raise eight or ten corpses in a single night; and he adds that he opened many graves, and refilled them again, with no assistance but his hands. He had not the courage of telling the whole truth in his written declaration; but he confessed to his medical attendant, M. Marchal, (de Calvi), the most repulsive part of this awful tale—viz., his preference for the remains of females, and his hideous propensity of satisfying sexual desires upon them. He was wounded when getting over the wall of the cemetery of Mont Parnasse, in Paris, brought to the hospital, and thus was unveiled this unheard-of train of disgusting acts.

The court-martial have not taken that view of the case which at first sight would have looked the most rational; and waiving altogether the possibility of monomania having impelled the man to these hideous deeds they looked upon the offence as a misdemeanor, and condemned him to one year's imprisonment.

Different opinions have been given in the medical journals as to which of the two kinds of mania exhibited was the first in existence—viz., the destructive, or the erotic. M. Marchal, the sergeant's medical attendant thinks the destructive prevailed: but M. Michea, a well-known mental pathologist, maintains that the second was, on the contrary, the strongest and only mania. The various circumstances mentioned by each of these gentlemen, to strengthen their respective positions, merely rest on the prisoner's own declaration; so that it would appear that no very strong case can be made on either side. Indeed, the whole series of these shocking occurrences might well be called in question, as it seems that no direct and conclusive evidence has been brought forward besides the man's own account. But assuming the latter as true, the existence of monomania can hardly be doubted, when we consider that a natural instinct was entirely set aside, that there was not the slightest prospect of gain, that the wish of visiting churchyards returned almost periodically, that the dangers incurred were entirely disregarded, that none of the vices which generally accompany depravity were present, &c. There was, besides, a melancholy disposition, a total insensibility to the agency of physical agents, (such as cold, rain, &c.,) during the paroxysm, and the extraordinary amount of muscular and nervous energy in the accomplishment of the acts, &c. All these considerations would tend to prove that this man was irresistibly impelled to such unheard of abominations.

This disgusting case recalls at once that form of mental aberration which reigned so extensively, about a century and a half ago, in the north of Europe, and known under the name of vampirism. It will be recollected that vampires were suffering under a sort of nocturnal delirium, which was often extended to the waking hours, during which they believed that certain dead persons were rising from their graves to come and draw their blood; hence arose a desire for revenge, and burial-places were disgracefully desecrated. Bertrand's case seems the very reverse of this; for we here see, not the dead rising to torment the living, but a man disturbing the peace of cemetries in the most horrible manner imaginable.—*London Lancet.*

